# Genetic determinants of hyaloid and retinal vasculature in zebrafish

**DOI:** 10.1186/1471-213X-7-114

**Published:** 2007-10-15

**Authors:** Yolanda Alvarez, Maria L Cederlund, David C Cottell, Brent R Bill, Stephen C Ekker, Jesus Torres-Vazquez, Brant M Weinstein, David R Hyde, Thomas S Vihtelic, Breandan N Kennedy

**Affiliations:** 1 UCD School of Biomolecular, and Biomedical Sciences, University College Dublin, Dublin 4, Ireland; 2 Electron Microscopy Laboratory, UCD Conway Institute, University College Dublin, Dublin 4, Ireland; 3 Center for Transposon Research. Department of Genetics, Cell Biology and Development. University of Minnesota, Minneapolis, MN 55455, USA; 4 Skirball Institute of Biomolecular Medicine. New York University School of Medicine, New York, NY 10016, USA; 5 Laboratory of Molecular Genetics, NICHD, NIH, Building 6B, Room 309, 6 Center Drive, Bethesda, MD 20892, USA; 6 Center for Zebrafish Research. Department of Biological Sciences. University of Notre Dame, Notre Dame, IN 46556, USA

## Abstract

**Background:**

The retinal vasculature is a capillary network of blood vessels that nourishes the inner retina of most mammals. Developmental abnormalities or microvascular complications in the retinal vasculature result in severe human eye diseases that lead to blindness. To exploit the advantages of zebrafish for genetic, developmental and pharmacological studies of retinal vasculature, we characterised the intraocular vasculature in zebrafish.

**Results:**

We show a detailed morphological and developmental analysis of the retinal blood supply in zebrafish. Similar to the transient hyaloid vasculature in mammalian embryos, vessels are first found attached to the zebrafish lens at 2.5 days post fertilisation. These vessels progressively lose contact with the lens and by 30 days post fertilisation adhere to the inner limiting membrane of the juvenile retina. Ultrastructure analysis shows these vessels to exhibit distinctive hallmarks of mammalian retinal vasculature. For example, smooth muscle actin-expressing pericytes are ensheathed by the basal lamina of the blood vessel, and vesicle vacuolar organelles (VVO), subcellular mediators of vessel-retinal nourishment, are present. Finally, we identify 9 genes with cell membrane, extracellular matrix and unknown identity that are necessary for zebrafish hyaloid and retinal vasculature development.

**Conclusion:**

Zebrafish have a retinal blood supply with a characteristic developmental and adult morphology. Abnormalities of these intraocular vessels are easily observed, enabling application of genetic and chemical approaches in zebrafish to identify molecular regulators of hyaloid and retinal vasculature in development and disease.

## Background

The retina is one of the most metabolically active tissues, with a higher rate of oxygen consumption than the brain [1]. Nourishment of the retina by a vascular network is critical for vision, and severe forms of human blindness including diabetic retinopathy, age-related macular degeneration and retinopathy of prematurity are linked with vascular abnormalities [2,3].

In most mammals, the adult retina is nourished by two independent circulatory systems, choroidal and retinal vessels. Choroidal vessels overlying the retinal pigmented epithelium (RPE) carry ~80% of retinal blood flow, and nourish the outer retina. The central retinal artery emanating from the optic nerve head carries the remaining ~20% of blood flow and nourishes the inner two thirds of the retina [4]. These retinal vessels develop intraretinal capillaries that ramify at the inner and outer plexiform layers [3,5]. Retinal vessels in mammals are not fenestrated and nourish the retina by transcytosis of nutrients, encapsulated in vesicle vacuolar organelles [6]. Unique to retinal and brain capillaries, pericytes directly contact the vascular endothelium, are enclosed by the basement membrane and have strong expression of smooth muscle actin conferring contractile function [5,7].

During mammalian eye development, the inner retinal vasculature is absent, and oxygenation of the retina is provided by choroidal and hyaloid vessels. The hyaloid vasculature is a complex of transient intraocular vessels comprising: i) the *vasa hyaloidea propia *(VHP), ii) the *tunica vasculosa lentis *(TVL) and iii) the *pupillary membrane *(PM). The VHP corresponds to the hyaloid artery entering the retina at the optic disc and branching anteriorly through the vitreous to the lens. The TVL is a capillary network cupping the posterior region of the developing lens, while the PM is an extension of the TLV that covers the anterior lens [8]. Hyaloid vessels undergo progressive regression as the retinal vasculature develops on an astrocyte scaffold [9,10]. There has been much debate as to whether the retinal vasculature forms by angiogenesis or vasculogenesis, though angiogenesis is now accepted [3,5,11]. Regression of the hyaloid vasculature by apoptosis and formation of the retinal vasculature by angiogenesis are synchronised processes. Failure of the hyaloid vasculature to regress is associated with ocular pathologies referred to as *persistent foetal vasculature*. Symptoms include severe intraocular haemorrhage, retinal detachment, cataracts and eventually blindness. Persistence of the hyaloid vasculature is accompanied by incomplete retinal vascularization, suggesting that overlapping signalling networks simultaneously control hyaloid regression and formation of the retinal vessels [8,12-14].

Although some genetic and environmental factors required for correct development of the retinal vasculature are known, the genetic pathways remain poorly defined [11]. A critical role for genetics in retinal vasculature development is exemplified by *Norrie *disease, an X-linked congenital syndrome characterized by persistence of the hyaloid vasculature and abnormalities in retinal vessels. This disease results from mutations in the *norrie disease protein *(NDP), a novel ligand for the frizzled-4 receptor that activates the canonical *Wnt *pathway [15]. Mutations in frizzled 4 cause *familial exudative vitreoretinopathy (FVER)*, a developmental disorder with abnormal retinal vascularization. In addition, Wnt7b expression on retinal macrophages is essential for apoptotic regression of hyaloid vessels in mice [14]. Indeed, Wnt, Frizzled and NDP are proposed as a new family of angiogenic factors [16].

In mammals, vascular endothelial growth factor-A (VEGF-A) plays a key role in the formation of the hyaloid vasculature, its regression and the formation of retinal vasculature. VEGF-A transcripts are detected in the developing lens as well as in the astrocyte scaffold accompanying the nascent retinal vessels [17,18]. In mouse, transgenic expression of VEGF-A isoforms from lens specific promoters, results in hyperplastic hyaloid vessels and abnormal patterning of retinal vasculature, with more diffusible forms of VEGF-A causing more severe phenotypes [19]. Lens crystallins are upregulated in persistent foetal vasculature, suggesting an unforeseen role in regulating retinal vascularization [20].

The amenability of zebrafish to high-throughput genetic and pharmacological screens *in vivo *provides novel opportunities to decipher vertebrate genes associated with vasculature defects and to identify lead drugs for therapeutic intervention [21-24]. These approaches are enhanced by the availability of the *Tg(fli1:EGFP) *transgenic line, which specifically expresses EGFP in blood vessels [25]. Genes required for trunk vasculature have been identified through characterisation of zebrafish mutants and morphants [26,27]. Subsequently, drugs which rescue a zebrafish model of lethal aortic coarctation *(gridlock)*, were identified in random small molecule screens [28]. However, extensive analysis of the morphology and genetic determinants of hyaloid and retinal vasculature in zebrafish has not been performed. This reflects difficulties in analysing these vessels in whole animals because of the refractive properties of the lens and the technical expertise required to dissect the minute larval eyes (~0.5 mm).

Here we develop methods to analyse the hyaloid and retinal vasculature in larval and adult zebrafish, and we document their development, morphology and ultrastructure. Using this framework, and screening zebrafish mutants and morphants, we identify a cohort of genes encoding extracellular matrix and cell surface proteins required for hyaloid and retinal vasculature development (Table 1).

**Table 1:** Mutant and morphants examined in the present study. Larval stages and number of specimens analysed are detailed in the table, together with a brief description of the overall phenotype and the morphology of the intraocular vasculature

**NAME**	**CATEGORY**	**AGE**	**n**	**GENERAL PHENOTYPE**	**HYALOID -RETINAL PHENOTYPE**
MAGP1	MORPHANT Microfibril associated glycoprotein 1	3 dpf	15	Dilated brain and caudal vessels. Irregular lumen of axial vasculature ***(Chen et al. 2006)***	Stagnated growth of hyaloid vasculature with less and thicker branches. Aggregation of vascular endothelial cells at the posterior lens
		5 dpf	15		
HS6ST2	MORPHANT heparan sulfate sulfotransferase 2	5 dpf	15	Over-lumenized vessels and defective branching in caudal vein plexus ***(Chen et al. 2005)***	Hyaloid vasculature with scarce and oversized branches that display an aberrant patterning.
Syn 2	MORPHANT Morphant of syndecan 2	5 dpf	6	Aberrant or absent sprouting in the intersegmental vessels ***(Chen et al. 2004)***	Sparse disorganized endothelial cells surrounding very small lens.
Sppl2b	MORPHANT Signal peptide peptidase like protein 2b	5 dpf	10	Erythrocyte accumulation in an enlarged caudal vein ***(Krawitz et al. 2005)***	NO PHENOTYPE
Mab21l2	MORPHANT Male abnormal 21 like-2 protein	5 dpf	5	Microphthalmia. Apoptosis in the developing retina and lens ***(Kennedy et al. 2004)***	Thicker vessels that cover only posterior part of the lens.
*obd*	VASCULAR MUTANT Plexin-D1 *out of bounds*	3 dpf	5	Mispatterning of the trunk intersegmental vessels (variable severity). ~10% of mutants die before 6 dpf ***(Childs et al. 2002; Torres-Vazquez et al. 2004)***	Mis-patterning of the hyaloid vessels (variable severity). Abnormal branching and extra interconnections obvious at 5 dpf. Atypical loops, tortuosity and increased number of vessels in the retinal vasculature of the adult.
		5 dpf	20		
		6 dpf	5		
		10 dpf	5		
		20 dpf	3		
		Adult	10		
*ace*	VASCULAR MUTANT FGF8 Hypomorph *acerebellar*	5 dpf	5	General CNS abnormalities. Absence of vessels in the dorsal brain. Abnormal heart development ***(Reifers et al. 1998; Reifers et al. 2000)***	NO PHENOTYPE
*arl*	LENS MUTANT Laminin alpha1 *arrested lens*	3 dpf	3	Abnormal eye development. Lens development halted at 24 hpf. Lack of any lens structure from 48 hpf. ***(Vihtelic et al. 2001; *** ***Vihtelic and Hyde 2002; *** ***Semina et al. 2006)***	Complete absence of hyaloid vessels in all stages analysed
		4 dpf	3		
		5 dpf	3		
		6 dpf	3		
*mgf*	LENS MUTANT/Unknown *margin affected*	4 dpf	7	Abnormal eye development from 3 dpf. Very small lens ***(Unpublished)***	At 4 dpf rudimentary hyaloid vasculature (fewer vessels and branches) on lens. At 5 dpf complete absence of hyaloid vasculature on small lens
		5 dpf	8		
*dsl*	LENS MUTANT/Unknown *disrupted lens*	5 dpf	5	Abnormal eye and lens development. ***(Vihtelic et al. 2001; *** ***Vihtelic and Hyde 2002)***	NO PHENOTYPE
		6 dpf	4		
		8 dpf	5		
*fe*	LENS-VASCULAR MUTANT/Unknown *fused eyes*	3 dpf	7	Synophthalmia. Abnormal/absent intersegmental blood vessels. ***(Unpublished)***	No hyaloid vessels on the lens
*plt*	LENS MUTANT/Unknown *platinum*	4 dpf	3	Defects in melanin pigmentation. Photoreceptor and RPE degeneration from 5 dpf. ***(Vihtelic et al. 2001;*** *** Vihtelic and Hyde 2002)***	Premature detachment of hyaloid vessels from the lens at 4 dpf. Less vessels and less patterning of the branches covering only posterior lens at 6 dpf.
		5 dpf	3		
		6 dpf	3		
*lop*	LENS MUTANT/Unknown *lens opaque*	6 dpf	4	Lens opacity ***(Vihtelic et al. 2005)***	NO PHENOTYPE

## Results

### Adult zebrafish have a random network of retinal vessels

**Figure 1:**
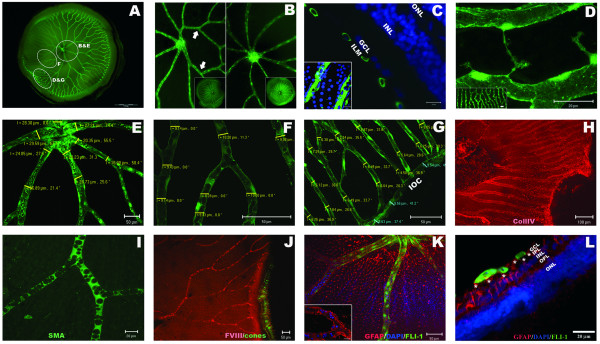
**Adult zebrafish have a complex system of retinal blood vessels**. **A: **Wholemount retina showing central major vessels that radiate into thinner vessels covering the entire inner surface of the retina. **B: **Example of disparity in branch number between left (5) and right (8) eyes. **C: **Transverse view of the blood vessels (green: Fli1-EGFP) overlaying the inner limiting membrane of the adult retina (blue: DAPI nuclear staining). Inset shows the same vessels overlying GCL nuclei in a flatmount preparation. **D: **At peripheral retinal regions, neighbouring vessels anastomose (inset), and elongated filopodia sprout from the capillaries suggesting active angiogenic remodelling. **E-G: **The diameter of vessels is thicker proximal to the optic disc and thinner peripherally. Numbers refer to the thickness of the vessel and the angle of measurement in reference to horizontal plane. **H-K: **Flatmount adult retinas immuno-labelled with retinal vasculature markers. **H: **collagen IV (red) stains the basal membrane of blood vessels. **I: **smooth muscle actin (SMA) stains vascular pericytes (green). **J: **Factor VIII labels endothelial cells (red). Cone photoreceptors label green as analysis was performed in *Tg(3.2TαCP:EGFP) *transgenic line [67]. **K-L: **Glial fibrillary acidic protein (GFAP) stains retinal vessels in adult zebrafish and a population of cells throughout the retina. GFAP (red), DAPI (blue) and fli1-EGFP (green). **K: **Flat-mounted retina, and **L: **Transverse view of the peripheral retina. Müller endfeet (asterisks) directly contact the endothelial cells (yellow co-staining). **Inset in K**: FITC channel turned off to highlight the GFAP reactivity of retinal vessels. *ILM: inner limiting membrane; GCL: ganglion cell layer; IPL: inner plexiform layer; OPL: outer plexiform layer; INL: inner nuclear layer; ONL: outer nuclear layer; IOC: inner optic circle*.

The morphology of the retinal vasculature in adult *Tg(fli1:EGFP) *zebrafish was characterised using fluorescent microscopy to directly visualise the vessels (Fig 1A–G and 1K–L). The retinal vasculature in wild type and albino animals was determined by immunohistochemistry and nuclear staining (Fig 1H–L and data not shown).

The inner limiting membrane of the zebrafish retina is covered by a complex system of blood vessels (Fig 1). The optic artery penetrates the retina posteriorly through the optic stalk and enters the vitreal space. At the optic disc, 4 to 9 main vessels ramify and spread over the inner surface of the retina (Fig 1A–B). The vasculature constitutes a membranous layer firmly attached to the vitreal interface of the adult retina. The vessels are in direct contact with the ganglion cell layer (Fig 1C) but do not form subretinal plexi. The main branches arborise radially from the optic disc eventually anastomosing with neighbouring capillaries before connecting to a circumferential vein (Fig 1D and 1G). This vein is the inner optic circle (IOC) which surrounds the retina at the cilliary marginal zone [29,30]. From 30 dpf to 22 months some of the thinnest capillaries show sprouting filopodia (Fig 1D) consistent with active angiogenesis [10,18]. The diameter of the vessels is ~26 ± 3 μm proximal to the optic disc, ~9.8 ± 1 μm at mid retina and reduces to capillary size of ~6.5 ± 0.8 μm at peripheral positions (Fig 1E–G). The IOC has a constant diameter of 9.5–12 μm. In contrast, branch point distances vary from ~30 to ~300 μm with no apparent relation to their position relative to the optic disc (Fig 1A).

The majority of adult retinas have 6–7 retinal vessels branching from the central artery (Table 2). The exact pattern of the vessels varies amongst individuals and an asymmetrical number of branches are observed between the left and the right eyes of ~80% of adult zebrafish analysed (Table 3). In ~9% of retinas that only have 4–5 main vessels (n = 45) two of these branches anastomose close to the optic disc, enabling arborisation of the entire retina (Fig 1B).

**Table 2:** Random numbers of retinal vessels branch from the optic disc. The number and patterning of the retinal vessels was examined in 150 retinas from ~100 adult wild type zebrafish. Most of the retinas (64%) exhibit 6 or 7 main branches radiating from the optic disc, although the number of vessels varies from 4 to 9. In some individuals (n = 21), the retina from the left eye was compared to the retina from the right, showing no apparent correlation between anatomical location and the number of main branches.

***Number of branches***	**4**	**5**	**6**	**7**	**8**	**9**
**Retinas **(n = 150)	7%	23%	39%	25%	5%	1%
**Left retinas **(n = 21)	10%	33%	23%	29%	0%	5%
**Right retinas **(n = 21)	0%	38%	38%	14%	10%	0%

**Table 3:** Asymmetrical number of main retinal branches in individual fish. Left and right retinas from individual zebrafish (n = 21) show differential number of main branches.

***DIFFERENCE IN NUMBER OF BRANCHES *LEFT VS RIGHT EYE**	**0**	**1**	**2**	**3**
**Percentage of fish **(n = 21)	19%	52%	24%	5%

Markers of retinal vasculature described in mammals are present on the zebrafish retinal vasculature (Fig 1H–J). Collagen IV marks the basal lamina of the vessels, smooth-muscle-actin stains retinal pericytes, and factor VIII labels the vascular endothelial cells [5,9,13]. To elucidate whether the retinal blood supply in zebrafish is associated with a glial scaffold comprised of astrocytes as occurs in humans and mice, glial-fibrillary-acidic-protein (GFAP) staining was performed [3,9]. Although we find no evidence of an astrocytic scaffold associated with the zebrafish retinal vasculature, we do find GFAP staining the retinal vessels in adult zebrafish (Fig 1K) and a population of cells throughout the retina. This GFAP staining is stronger at the optic disc and the peripheral retina. Morphology and immunolabelling identifies them as Müller cells and not astrocytes or microglia (Fig 1K–L). These GFAP-positive Müller cells span the entire retinal thickness and at the ganglion cell layer, the Müller endfeet come into contact with the basal lamina of the retinal vessels, forming projections which directly contact the vascular endothelial cells (Fig 1K–L). Analysis of retinas from *Tg(gfap:EGFP) *transgenic animals (Additional file 1), which express EGFP under control of a glial fibrillary acidic protein (GFAP) promoter, confirms the direct contact between the Müller cells and the vessels. When the vascular layer is dissected from the retina, the Müller endfeet remain attached to the vessels revealing a tight vascular-glial interaction (Additional file 1C).

### Dynamic development of the hyaloid-retinal vasculature in zebrafish

**Figure 2:**
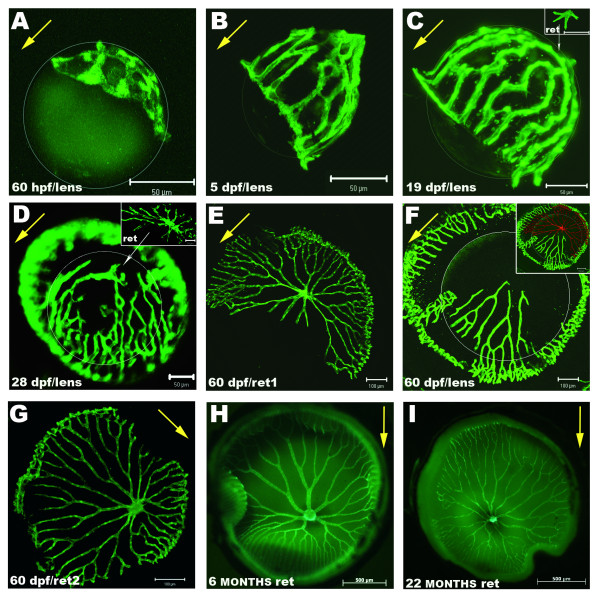
**Dynamic development of the hyaloid and retinal vasculature in zebrafish**. Shown are fluorescent images of blood vessels on lenses and wholemount retinas dissected from *Tg(fli1:EGFP) *zebrafish. **A: **First intraocular vessels are detected at 60 hpf surrounding and attached to the forming lens. **B: **This vasculature evolves quickly and at 5 dpf covers the lens from the optic disk to the IOC. **C: **At 19 dpf an intricate network of hyaloid vessels branches around the lens. Some vessels at the posterior lens have lost contact and are attached to the retina (inset). **D: **At 28 dpf, detachment of the vessels from the lens has progressed anteriorly from the central region and extensive vasculature is found on the inner retina. **E: **Retina and **F: **lens from a 60 dpf zebrafish. In this specimen some vessels are attached to the lens although most of them are found on the retina. **Inset in F: **Overlay of the retina from **E **pseudo-coloured in red, and the lens from **F **depicts the complementing network of vessels. **G: **Retina of another 60 dpf zebrafish with the complete vasculature overlying the inner retina. **H: **Typical vascular pattern of intraocular vasculature in a 6 month old fish. **I: **Inner retina of a 22 month old senescent zebrafish showing slightly thinner and more fragile vessels. White circumferences demarcate the lens in A-F. Yellow arrows point from posterior to anterior lens in A-D&F and from dorsal to ventral retina in E&G-I. *Scale bars: 50 μm in A-D; 100 μm in E-G and 500 μm in H-I*.

To characterise the development of the retinal blood supply in zebrafish, eyes of *fli1-EGFP *transgenic fish from 24 hpf to 22 months old were analysed. By 48 hpf, some EGFP positive cells are present between the lens and the retina ***(data not shown)***. These endothelial cells give rise to the first hyaloid vessels, distinguishable by 2.5 dpf as a rudimentary vasculature tightly attached to the lens (Fig 2A). At 5 dpf, the vessels are organized in a hemispherical basket embracing the lens from the central optic disc to the peripheral IOC (Fig 2B). As this vasculature grows around the lens, the vessels rapidly branch and at 19 dpf have formed an intricate network (Fig 2C). Numerous filopodia project from these vessels indicating dynamic patterning by angiogenesis [18].

At 15 dpf, the vitreo-retinal vasculature gradually becomes more attached to the retina loosening its contact to the lens (Figs 2C–D). This detachment initiates at the centre of the posterior lens and moves anteriorly (Fig 2C–D). Detachment from the lens and association to the retina is progressive, as vessels missing on the dissected lens complement those found on the corresponding retina (inset in Fig 2F). The age at which this transition occurs is variable and reflects the diverse rates of larval growth. At 60 dpf, juvenile zebrafish retinas show an elaborate vasculature largely identical to adults (Fig 2E–G). This movement of the hyaloid vessels to form the mature retinal vasculature contrasts with mammals where remodelling by hyaloid regression and retinal angiogenesis is necessary (Fig 2C–F) [8,9]. From 2 to 18 months the morphology of the retinal vasculature looks similar. However in senescent fish (> 18–20 months) the retinal vessels progressively become thinner and more fragile (Fig 2I), consistent with an age-related loss of retinal vasculature function [31].

### The zebrafish retinal vasculature features pericytes junctional complexes and vesicle vacuolar organelle

**Figure 3:**
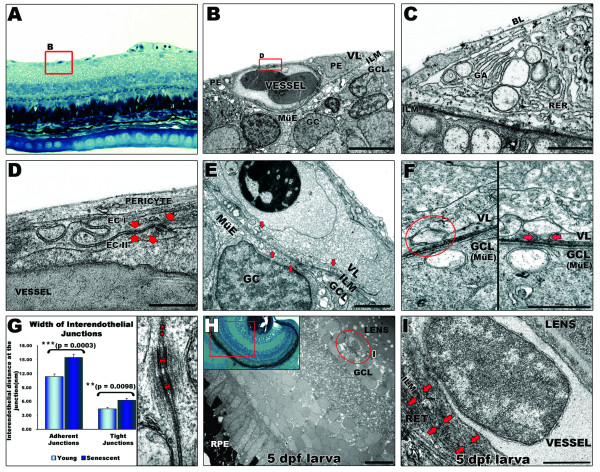
**Ultrastructural analysis of the inner retina blood supply in zebrafish**. **A: **Light microscopy image of blood vessels (highlighted by a red square) overlying the inner limiting membrane (ILM) of the retina in adult zebrafish. *40× magnification*. **B: **Ultrastructure of capillary. The basal lamina (BL) is in contact with the ILM enclosing pericytes (PE) and the vascular endothelium. *Scale bar: 5 μm*. **C: **Typical structural features of pericytes, *e. g. Golgi apparatus *(GA), rough endoplasmic *reticulum *(RER) and large membrane bound vesicles. *Scale bar: 1 μm*. **D: **Vitreal space lined by pericyte overlying endothelial cells (EC) which display interdigitating junctional complexes (arrows). *Scale bar: 500 nm*. **E: **Multiple vesicles (arrows) from 20 to 250 nm contacting the inner limiting membrane of the retina indicate active interaction between the vessels and the ganglion cell layer (at the Müller endfeet). *Scale bar: 1 μm*. **F: **Vascular (top) and ganglion cell layer interface (bottom). A vesicle (left panel) apparently separated from the cell membrane, fuses with the cell membrane when the section is tilted by 43° (right panel) suggesting transcellular transport. *Scale bar: 500 nm*. **G: **Interendothelial junctions are significantly more open in the senescent fish. Distance between endothelial cells at tight (asterisks) and adherent (arrows) junctions were measured in different peripheral and central areas of retinas from senescent and young adult fish. *Error bars: sem*. **Inset in H: **Histology of a 5 dpf zebrafish eye. *40× magnification*. **H: **EM image of a 5 dpf larval eye illustrating the relationship of hyaloid vessels (encircled by a red line) to both the lens and the retina. *Scale bar: 10 μm*. **I: **Higher magnification of the hyaloid vessel confirms its tight attachment to the lens and looser contact to the retina (arrows). *Scale bar: 1 μm. MüE: Müller cell endfeet; GC: ganglion cell; GCL: ganglion cell layer; VL: vascular layer; RPE: retinal pigmented epithelium; RET: retina*.

The ultrastructure of the zebrafish vitreo-retinal vessels was examined to elucidate cellular interactions with the lens and retina (Fig 3). Sections of retinas from adults confirm the presence of a vascular system in direct contact with the inner limiting membrane and the ganglion cell layer (Fig 3A). Vascular endothelial cells are interconnected forming interdigitating junctional complexes (Fig 3B–D). Multiple mature pericytes with Golgi networks and vacuoles (Fig 3B–C) are found associated with the vessels in all young and senescent specimens, but not in the larvae (Fig 3H). The pericytes are located between the vascular endothelium and the basal lamina of the retinal vessels in direct contact with both (Fig 3B–C), as observed in mammalian retinas [5,32]. At the inner limiting membrane, numerous vesicles ranging from ~20–250 nm are coupled to the cell membranes at both the vascular and the ganglion cell sides (Fig 3E). These vesicles fuse with the plasma membranes (Fig 3F) suggesting nourishment by active transcellular transport, equivalent to the vesicle vacuolar organelles (VVO) observed in mammalian retinas [6].

In senescent fish, the inner surface of the retina is also vascularised and there are no apparent differences in the number of pericytes or presence of VVOs compared to younger fish. The senescent retinas are distinguished by the thinner diameter (~19 ± 3 μm) of the vessels proximal to the optic disc compared to the younger fish (~26 ± 3 μm), an observation supported by fluorescence microscopy (Fig 2). Detailed morphometric analysis shows a significant increase in the width of the tight and adherent interendothelial junctions in senescent zebrafish (Fig 3G) resembling that observed in age-related degeneration of mammalian retinal vasculature [33-35].

There is no vitreal space in 5 dpf zebrafish larvae [36], and consequently the intraocular vasculature is in direct contact with the lens and retina (Fig 3H). The attachment of the vessels to the retina is not as comprehensive as with the lens (Fig 3I) an observation supported by the visualisation of the vessels on the lens after dissection (Fig 2). The ultrastructure of the vessels in larval eyes confirms close association of the vascular basal lamina and the lens via fused membranes (Fig 3H–I). In contrast to the mammalian TVL (part of hyaloid vasculature covering the posterior lens), no fenestrations are found on the zebrafish vascular interface contacting the lens, instead VVOs indicative of transcytosis are present [8,37].

### ECM and cell membrane proteins required for hyaloid-retinal vasculature

**Figure 4:**
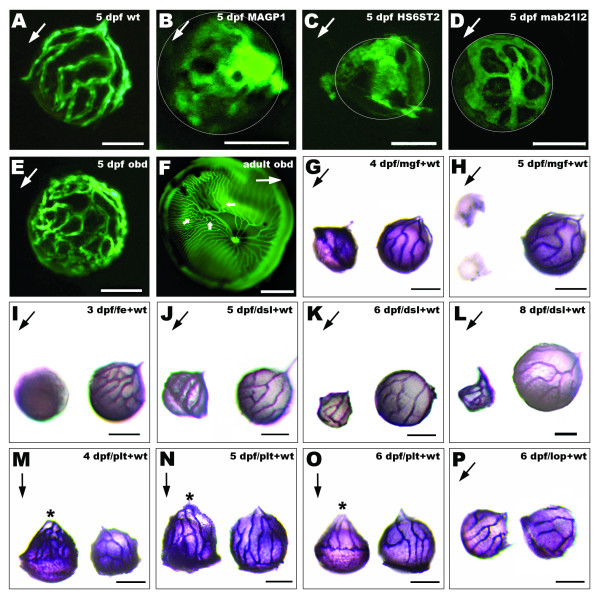
**Genes required for hyaloid and retinal vasculature development**. **A-E: **Fluorescent images of *fli1-EGFP hyaloid vessels *on dissected lenses. **A: **Characteristic pattern of hyaloid vasculature attached to the lens of 5dpf wild type fish. **B: **Stagnated growth of hyaloid vasculature with less and thicker branches in MAGP1 morphants, and aggregation of vascular endothelial cells at the posterior lens. **C: **Aberrant oversized branches and lack of patterning in hyaloid vasculature of 5 dpf *HS6ST2 *morphants. **D: **hyaloid vessels in 5 dpf *mab21l2 *morphants are thicker and poorly patterned. **E: **Poor patterning and abnormal branching of the vessels attached to the lens of 5 dpf *obd *mutants **F: **Wholemount retina showing the retinal vasculature of an adult *obd *mutant exhibiting atypical loops (small arrows) and increased number of vessels. **G-P: ***Alkaline phosphatase staining of hyaloid vasculature on lenses from mutant (left) and wild type larvae (right)*. **G-H: **Lens from *mgf mutants *is reduced in size and shows only residual unorganized vascular tissue at 4 dpf and no vessels at 5 dpf. **I: **Lens from *fe *mutants is slightly smaller and has no hyaloid vessels at 3 dpf. **J-L: **Lens from *dsl mutants *are much smaller but the hyaloid vasculature displays no apparent abnormalities at 5–8 dpf. **M-O: **Hyaloid vessels of *plt *mutants are loose at the back of the lens (asterisks) and the vascular pattern gradually becomes less intricate. **P: **Vasculature attached to lens of 4–6 dpf *lop *mutants is similar to vessels on wild type lens. Arrows point from posterior to anterior lens in all panels expect in 4F, where it points from dorsal to ventral retina. *Scale bars: 50 μm in all panels except F, 500 μm*.

We screened genes known to have caudal and/or intersegmental vascular phenotypes, for defects in hyaloid and retinal vessels. In both MAGP1 and HS6ST-2 morphants, the development and patterning of the hyaloid vasculature appears arrested, displaying defects in angiogenic remodelling and failing to coalesce into defined branches (Fig 4B–C). Lens vessels in morphants of the microfibril-associated glycoprotein 1 (MAGP1) have a phenotype (Fig 4B) similar to related abnormalities described for the trunk vasculature [38]. Morphants of MAGP1 exhibit an aggregate of vascular endothelial cells at the posterior lens and an extremely reduced number of branches that appear thicker, poorly patterned and stagnated compared to hyaloid vasculature of wild type larvae or control morphants (Fig 4A–B). Morphants of the extracellular matrix regulator, heparan sulfate sulfotransferase 2 (HS6ST-2) have caudal vein defects being overlumenized, obstructed and displaying less branching [39]. In the HS6ST-2 morphants vascular endothelial cells do proliferate to expand across the lens but fail to remodel to form a defined network of hyaloid vessels (Fig 4C). Morphants of syndecan 2 (Syn 2), a cell surface heparan sulfate proteoglycan essential for angiogenic sprouting in the trunk [40], completely lack vessels or their precursors on the lens (Table 1 and data not shown). In contrast no significant abnormalities of the vitreoretinal vasculature were observed in morphants for the signal peptide peptidase homologue (Sppl2) although it is necessary for caudal vein formation (Table 1 and data not shown) [41].

**Figure 5:**
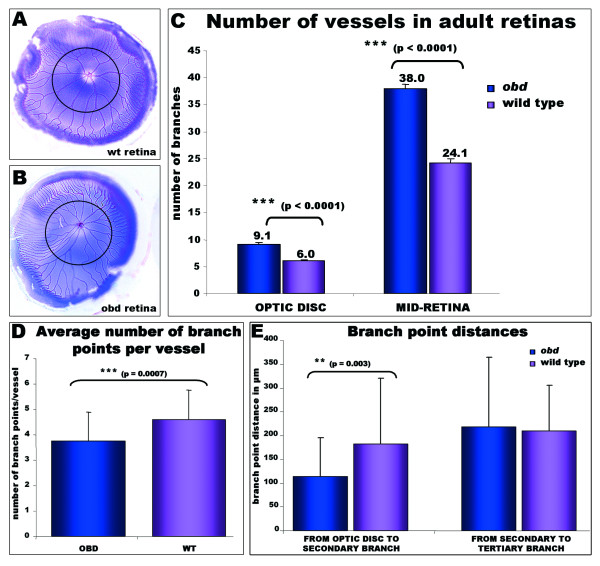
**Adult-onset retinal vasculature phenotypes in Plexin D1 mutants**. **A-B: **Pseudo-coloured adult retinas from (A) wild type and (B) *obd *adult fish to facilitate quantification of the vessel branches. A black circumferential line demarks half the distance between the optic disc and the annular peripheral vein at different retinal regions (MID- retina). **C: **The number of main branches radiating from the optic disc and the number of vessels crossing the mid-retina are higher in *obd *mutants (n = 18) than in wild type retinas (n = 24). **D: **The average number of branch points per vessel is significantly decreased in *obd *(n = 5) versus wildtype (n = 5) retinas. The number of branch points per 9 randomly chosen vessels in each retina was counted from outside in. To avoid bias, the nearest vessel was chosen every 40° of retinal circumference, starting from the most ventral poin. **E: **A significant reduction in the distance from the optic disc branches to the secondary branches, but not to the tertiary branches, is observed in *obd *vessels (n = 40) versus wildtypes (n = 22).

The plexin D1 receptor is mutated in *out of bounds *(*obd*) mutants resulting in patterning defects of intersegmental vessels [27,42]. In *obd *larvae, patterning defects of the hyaloid vessels are subtle at 3 dpf but by 5 dpf the vessels distinctly form abnormal branches and extra interconnections (Fig 4E). The phenotype is variable in juvenile (~30 dpf) where the hyaloid vasculature is often undistinguishable from wild type. However, in adult *obd *zebrafish increased tortuosity of the retinal vasculature is consistently observed, with highly contorted looping not found in wild type retinas (Fig 4F and Additional File 2). Compared to wild types, adult *obd *retinae have a significantly higher number of vessels at the mid-retina (Fig 5C and Additional File 2). The *obd *phenotype is not a result of more frequent vessel branching across all of the retina, as the average number of branches per vessel is not increased, but in fact is significantly decreased (Fig 5D). The higher number of retinal vessels in *obd *mutants stems from a significantly increased number of primary branches radiating from the optic disc (Fig 5C) and a significantly reduced distance to the secondary branch (Fig 5E).

### Lens regulators required for hyaloid vasculature development

The intimate association between the developing hyaloid-retinal vasculature and the lens suggests that lens-derived signals may regulate vessel development. Therefore we analysed the intraocular vasculature of several mutants characterised by defects in lens development.

*Arrested lens (arl) *mutants have a causative mutation in the gene encoding laminin alpha 1 [43-45]. In *arrested lens (arl) *mutants only a rudimentary lens vesicle is observed at 48 hpf which disappears by 60 hpf [43]. We observe that in the absence of laminin alpha 1, a complete loss of intraocular vasculature results at 3–6 dpf (Table 1 and data not shown). *Margin affected (mgf) *mutants exhibit small eyes and lens opacity at 4 dpf. At this stage *mgf *mutants exhibit disorganised vascular tissue attached to the lens, with fewer vessels and fewer branches than the wild type larvae (Fig 4G). At 5 dpf, *mgf *mutants have only a vestigial lens with no vasculature (Fig 4H). Similarly, *fused eyes *(*fe) *mutants have smaller lenses with no associated vascular structures (Fig 4I). Not all lens mutants present defects in the hyaloid vessels. Lens opaque mutants *(lop) *exhibit lens opacity but have a hyaloid vasculature indistinguishable from wild type (Fig 4P)[44]. Furthermore the loss of vessel development in *mgf *and *fe *mutants is not simply due to the smaller lens, as *disrupted lens *(*dsl) *mutants [43,44], have quite normal hyaloid vasculature at 3–8 dpf despite stunted lens growth (Fig 4J–4L). In addition we identify mutants with normal lens size that exhibit defects in vessel patterning. *Platinum *(*plt) *mutants display a subtle ocular phenotype recognisable after 4 dpf by small pupils and pale pigmentation (Vihtelic, unpublished data). *plt *lenses are normal in size at 5 dpf but ~20% smaller than wild type at 6 dpf (Fig 4M–O). Although intraocular vessels are present in the mutant they prematurely detach from the posterior lens at 4 dpf (Fig 4M–N). At 6 dpf, the *plt *mutant vessels are reduced in number, have less patterning of branches and cover only the posterior half of the lens (Fig 4O).

Using antisense morpholinos we determined a novel role for the *mab21l2 *gene in hyaloid vasculature development. Mab21l2 morphants display retinal and lens defects [46]. At 5 dpf, the hyaloid vessels in *mab21l2 *morphants are thicker than wild types, and their growth is retarded to only cover a small portion of the posterior lens, (Fig 4D).

## Discussion

**Figure 6:**
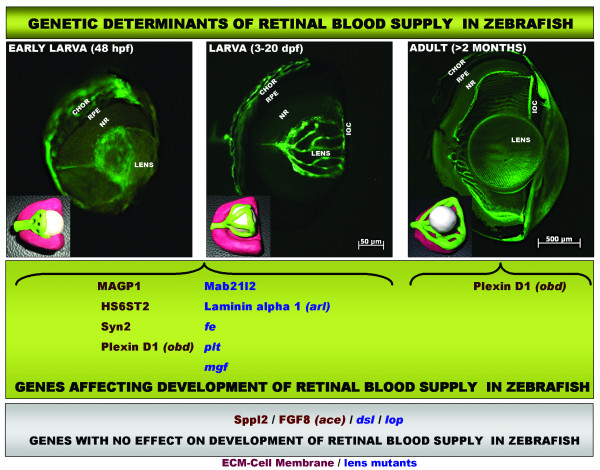
**Model demarking the stages of retinal vasculature development and genetic determinants in zebrafish**. Shown are fluorescent micrographs of partially dissected eyes from *Tg(fli1:EGFP) *fish showing the hyaloid and retinal vasculature in larvae and adult. Hyaloid vessels first appear attached to the back of the lens at 48 hpf and grow rapidly to reach the front of the lens at 3 dpf. In the adult, vessels are found associated with the inner surface of the retina. Insets are 3D models of this process where vessels have been coloured in green, retinas in pink and lens in white. Genes identified in our analysis to affect retinal vasculature development at different stages are boxed in green and genes unrelated to retinal vessels development are boxed in grey. ECM genes: red; lens/retina genes: blue. *CHOR: choroidal vasculature; RPE: retinal pigmented epithelium; NR: neuro-retina; IOC: inner optic circle*.

We describe the cellular and ultrastructural morphology of the hyaloid and retinal vasculature in zebrafish from early development through senescence and identify genes encoding extracellular matrix and cell surface proteins that are required for their development (Fig 6).

### Comparison of human and zebrafish retinal vasculature

Zebrafish provide a useful model to analyse the genetics and pharmacology of human retinal vasculature. In both humans and zebrafish; i) the primitive retinal vasculature branches by angiogenesis from the central retinal artery, ii) there is an initial hyaloid vasculature tightly associated with the lens and later a retinal vasculature tightly associated with the Müller endfeet in the ganglion cell layer, iii) the retinal vasculature is enriched in pericytes enclosed by the vessel basal lamina and iv) nourishment is likely mediated by subcellular vesicle vacuolar organelles exchanged between the vessels and retina.

However, several features distinguish developing human and zebrafish retinal vasculatures. An avascular region equivalent to the cone-enriched fovea of mammals is not present in adult zebrafish. In most mammals, the primary retinal vessels branch to form intraretinal capillaries that form plexi within the inner and outer plexiform layers [3,5]. These intraretinal plexi are not present in adult zebrafish, probably because the thinner zebrafish retina (~150 μm) can be nourished by the surface retinal and choroidal vessels. A similar phenomenon occurs in some mammals in which the entire retina is nourished only by the choriocapillaris [5,8]. The coupled hyaloid regression and retinal angiogenesis observed during human retinal vasculature development is not observed in zebrafish. Instead, the vessels gradually move away from the lens onto the retina as the vitreous forms. It is likely that the closer proximity of the retina and lens in zebrafish means that re-modelling rather than re-growth of the vessels is sufficient.

A hallmark of retinal vasculature development in mammals is the appearance during hyaloid regression of astrocytic networks that act as a scaffold and signalling source for the forming retinal vasculature [3,9]. We find no evidence of an astrocytic scaffold associated with the developing retinal vasculature in zebrafish. The scaffold is presumably unnecessary, as coupled hyaloid regression and retinal angiogenesis is not required. Nevertheless, we do find that the retinal vessels in zebrafish contact radial Müller glial cells. Perhaps these Müller cell-retinal vasculature interactions regulate retinal neovascularisation as they do in mammals under hypoxic conditions [47]. Additionally, signals emanating from zebrafish retinal vessels after injury have been reported to control reprogramming of the multipotent Müller cell lineage to produce a niche of stem cells that regenerate the injured retina [48].

### ECM and Cell Surface Factors Required for Retinal Vasculature Development

We find several cell surface and extracellular matrix (ECM) proteins required for hyaloid and retinal vasculature development. Some ECM components are present in all vessels and some are tissue-specific. The ECM provides structural support and acts as a signalling centre regulating development and repair of blood vessels [49].

Within the ECM, we identify a critical role for the gene encoding microfibril associated glycoprotein 1 (MAGP1) in retinal vasculature development. MAGP1 is known to be expressed in primitive vessels that first form on the posterior lens [38]. Consistently, in the absence of MAGP1, the growth of the hyaloid vasculature is stagnated and fails to form the characteristic wild type network of hyaloid vessels. Microfibrils provide elasticity to blood vessels and MAGP1 binds with fibrillin, a major component of mircofibrils [38]. Previously, MAGP1 has been reported to control vessel development in the trunk via integrin signalling [38]. Similar ECM-integrin signals presumably operate in the hyaloid vasculature. We also determine that defects in the ECM complement of heparan sulfate proteoglycans can result in defective vitreo-retinal vasculature development. Heparan sulfate proteoglycans on the cell surface and in the ECM are key regulators of growth factor signalling and pericyte recruitment during angiogenesis [50,51]. Impairment of this activity likely underlies the hyaloid vasculature defects in *HS6ST-2 *and *syndecan 2 *morphants. Heparan sulfate sulfotransferase 2 (HS6ST-2) differentially sulfates heparan sulfate proteoglycans conferring tissue-specificity to the extracellular matrix [39]. HS6ST-2 is expressed in the developing eye at 24 hpf and loss of its sulfotransferase activity results in a conglomeration of endothelial cells around the posterior lens that fail to form a normal branching network. Likewise, we find that loss of *syndecan 2*, a cell-surface heparan sulphate proteoglycan, results in a rudimentary lens lacking vitreoretinal vasculature, similar to the angiogenic sprouting defects reported for *syndecan 2 *in the zebrafish trunk [40]. Lastly, we find that the plexin D1 receptor expressed on the cell surface of endothelial cells is required for normal vascular patterning of the hyaloid vasculature. In addition, zebrafish plexin D1 mutants [42] survive to adulthood, unlike the knockout mice [52], enabling us to observe retinal vasculature phenotypes characterised by superfluous vessel branching and vessel tortuosity. The signalling networks accounting for the hyaloid and retinal vasculature abnormalities in the absence of plexin D1 remains to be determined. Plexins are receptors for semaphorins and are necessary for axonal path-finding and vascular patterning during development [53]. Indeed, plexin D1 has different roles in different vascular beds. In the trunk, where stereotypical vessel assembly normally occurs, disruption of semaphorin 3-plexin D1 signalling results in the intersomitic vessels launching from irregular positions, following aberrant paths of migration and interconnecting ectopically [27,42]. In the caudal plexus, which displays a variable vascular architecture, defects in vessel remodelling are observed upon disruption of plexin D1 signalling [27].

Although we identify MAGP1, HS6ST2, syndecan 2 and plexin D1 as genetic determinants of the hyaloid/retinal vasculature, they are generic determinants of vasculature. Evidence that the hyaloid/retinal vasculature has unique gene combinations underlying its development initially comes from our findings that no hyaloid vasculature defects are observed in *FGF8 *or *sppl2 *mutants, despite their fundamental role in vascular development of the brain and heart, and caudal vein, respectively [41,54,55]. We hypothesised that lens or retinal factors may specifically regulate retinal vasculature patterning. Development of hyaloid vessels tightly attached to the lens, followed by progressive attachment to the retina suggests key roles for secretory or cell surface signals emanating from the lens and retina. Further *proof-of-principle *is evident from experiments where lens expression of *norrie *transgenes partially rescues retinal vasculature defects in knockout mice [56]. Thus, we examined zebrafish with genetic defects in lens and retinal development for associated defects in the hyaloid/retinal vasculature.

### Retinal and Lens Factors Required for Retinal Vasculature Development

Our analyses demonstrate that *laminin alpha 1, mgf, fe, plt *and *mab21l2 *genes are required for hyaloid and retinal vascular development. Laminin alpha 1 is a glycoprotein constituent of basement membranes in ocular tissues. Loss of laminin alpha1 has recently been shown to result in a complex ocular phenotype including loss of lens structures and a disorganized ganglion cell layer [45]. Consistent with the published data reporting the failure of vascular endothelial cells to differentiate into capillaries, we find no hyaloid vasculature in *arl *mutants. *Mab21l2 *genes are highly conserved in vertebrates but have unknown intracellular function [46]. We find a role for *mab21l2 *in hyaloid vessels patterning as the vessels in *mab21l2 *morphants only cover the posterior lens, are thicker and have a less elaborated pattern. This is consistent with a proposed role for *mab21l2 *in cell proliferation [57]. Three other genes *fe, mgf *and *plt*, which remain to be cloned and fully characterised are also required for retinal vasculature development. Notably, *mab21l2, mgf *and *plt *appear to have a specific role in hyaloid vasculature formation as no defects in the trunk or gut vasculature are associated with these genes. Cloning and functional characterisation of these genes will help to define the networks controlling hyaloid vasculature formation.

### Zebrafish models of human retinal vasculature disease?

In addition to the identification of genetic determinants, zebrafish may also be used to create models of human hyaloid/retinal vasculature disease. Diabetic retinopathy, age-related macular degeneration and retinopathy of prematurity are prominent vascular abnormalities resulting in human blindness [2,3].

As *retinopathy of prematurity (ROP) *is a developmental disorder, it is logical to think that this leading cause of childhood blindness could be modelled in zebrafish. In ROP, premature infants are placed into high oxygen leading to cessation of hyaloid vessel regression and growth of nascent retinal vessels. Upon return to normoxia the infant retina is hypoxic leading to abnormal neovascularization which eventually leads to retinal detachment [12]. Our results, however, demonstrate that zebrafish cannot model this disease as they do not undergo overlapping hyaloid vessel regression and retinal vessel angiogenesis.

Diabetic retinopathy and age-related macular degeneration are adult-onset diseases characterised by leaky, dilated vessels and unwanted growth of new blood vessels into the vitreous. A good animal model to investigate the pathogenesis of vasculoproliferative retinal disease does not exist [58,59]. Adult zebrafish may fill this void as our results show their retinal vasculature to share many similarities with humans. These include pericytes and junctional complexes, cellular hallmarks which become perturbed in human disease [32,35,60].

## Conclusion

We present a framework documenting the morphology and ultrastructure of hyaloid and retinal vasculature development in zebrafish. We also identify an initial cohort of genes required for normal formation of these vasculatures. These results provide a foundation to utilise genetic and pharmacological approaches in zebrafish, to understand the development and diseases of hyaloid/retinal vasculature.

## Methods

### Zebrafish maintenance and strains

Zebrafish (*Danio rerio*) were maintained according to standard procedures on a 14-h light/10-h dark cycle at 28°C. Embryos were obtained by natural spawning and developmental stages established by time and morphological criteria [61]. *Tg(fli1:EGFP), Tg(gfap:EGFP) *and albino *(alb4) *lines were obtained from ZIRC [62].

### Morphants and mutants

Details of the morpholinos and mutants analysed are presented in Table 1. All morpholinos, including their sequences and specificity have previously been published in terms of trunk vasculature or eye size [38-41,46]. In brief, 6 ng of *MAGP1*, 8 ng of *HS6ST2*, 2 ng of *Syn2*, and 4 ng of *sppl2b *morpholinos and respective controls were injected at the 1–4 cell stage into *Tg(fli1:EGFP) *embryos using standard techniques [63]. Co-injection of a p53 morpholino at 1.5 times the concentration of the vascular-associated morpholino was used to reduce toxicity [64]. Injected embryos with viability greater than 50%, were incubated at 30°C for 3–5 days, and scored for the published tail vasculature phenotype [65]. *Mab21l2 *(antisense-1) and control morpholinos [46] at 2.5 mg/ml in Danieau buffer were injected into single-cell *Tg(fli1:EGFP) *embryos and incubated at 28°C for 5 days. Lens mutants (*obd, arl mgf, dsl, fe, plt and lop*) were selected based on described and unpublished phenotypes (Table 1). *Ace *mutants were obtained from Prof. Michael Brand (Biotechnological Center of the Technische Universität Dresden, Germany).

### Retina/lens dissection and preparation

Larvae and adult fish were euthanised with a lethal dose of benzocaine. Whole larvae (0–30 dpf) and juveniles (30–60 dpf) were fixed in 4% paraformaldehyde (PFA) in PBS for 2 hours at 4°C. The cornea was removed to facilitate retina and lens dissection. For adult fish (greater than 2 months old), eyes were enucleated and fixed in 4% PFA at 4°C (2 hours – overnight). After removal of the cornea and lens, retinas were dissected from adult eyes and fixed for another 15 min at room temperature before further processing. Retinas were flat mounted onto glass slides or directly embedded in 15% methylcellulose before transfer to depression slides for transmitted light microscopy or to coverslip glass-bottom Petri dishes for confocal microscopy. Lenses were embedded in 15% methylcellulose before microscopy. Vessels were visualised under brightfield, epi-fluorescence and confocal microscopes (Zeiss Lumar V12 stereo microscope, Olympus SZX16 stereo zoom microscope, Zeiss UV510 META LSM confocal microscopy system).

### Immunohistochemistry

Retinas were gradually dehydrated in methanol, rehydrated in PBS, permeabilised with PBS/0.5% Triton ×100 (PBT) for 30 minutes at RT and blocked in PBT/10% goat serum for 30 minutes at 4°C. Retinas were incubated overnight with the primary antibody (1:100 – 1:200) in PBT/10% goat serum at 4°C. Primary antibodies were: collagen IV (Chemicon International), smooth muscle actin (DakoCytomation), FVIII (DakoCytomation), and GFAP (Sigma). After washes the retinas were incubated with cy2- and cy3- conjugated secondary antibodies (Jackson Immunoresearch) diluted 1:200 in PBT/5% goat serum, for 2 hours at RT. The adult retinas were flat-mounted on glass slides and photo-protected with Vectashield (Vector) prior to confocal microscopy. Staining results were confirmed using primary antibodies diluted 1:100 followed by detection with peroxidase labelled secondary antibodies and colorimetric development reactions (DAB kit, Vector).

### Alkaline phosphatase staining

Staining of the endogenous alkaline phosphatase (AP) activity of hyaloid vessels was performed as previously described [66]. Briefly, larvae were fixed in 4% PFA overnight at 4°C and then rinsed with PBS. Eyes were pierced with a tungsten micro-needle and larvae transferred to 100% methanol for 2 hours at 4°C. Larvae were rehydrated gradually in PBS, rinsed in PBS/1% Tween-20 and treated for 30 minutes at RT with NTMT-alkaline phosphate buffer (pH = 9.5). NBT (0.4 mg/ml) and BCIP (0.19 mg/ml) were added and the staining was carried out in darkness at RT. Stained larvae were fixed for 2 hours in 4% PFA at 4 C. Lenses were dissected and prepared for microscopy as described above.

### Ultrastructural analysis

For transmission electron microscopy, whole eyes from adult (n = 3) and senescent (n = 3) fish were fixed in a mixture of 4% PFA and 2.5% glutaraldehyde diluted in 0.1 M Sorensen phosphate buffer (pH = 7.3) at RT. After 2 hours, retinas were dissected in the fixative and left overnight at RT. Whole 5 dpf larvae (n = 3) were directly fixed overnight using the same conditions. All samples were post-fixed in 1% osmium tetroxide in 0.1 M Sorensen phosphate buffer (pH = 7.3) for 1 hour at RT, dehydrated in ascending concentrations of alcohol to 100% and embedded in Epon resin using standard methods. Survey, semi-thin (1 μm) sections were examined by light microscopy where areas of interest were identified for ultrastructural analysis. Ultra-thin sections (80 nm) were cut using a diamond knife and a Leica UC6 ultramicrotome, picked up on 200 mesh copper grids and contrasted with uranyl acetate (20 mins) and lead citrate (10 mins). Sections were examined in a Tecnai 12 BioTwin transmission electron microscope (FEI Electron Optics) using an acceleration voltage of 120 Kv and an objective aperture of 20 μm. Digital images at various magnifications were acquired with a MegaView 3 camera (Soft Imaging Systems) and specimen tilting experiments were facilitated using the CompuStage alpha tilt features of the microscope.

## Authors' contributions

YA carried out the morphological time-course analysis of zebrafish hyaloid/retinal vasculature, characterised the phenotypes in most of the mutants and morphants and contributed to the ultrastructural analysis. MLC generated the *mab21l2 *morphants and helped characterise their hyaloid vasculature. DCC performed the ultrastructural analysis. BRB and SCE generated the vascular morphants. JTV and BMW provided the *obd *mutants and comments on phenotypes. TSV and DRH generated the lens mutants. BNK conceived, designed and supervised the study, and provided major contributions in drafting and writing the manuscript. All authors read and approved the final manuscript.

## Supplementary Material

Additional File 1 **Physical interaction of Müller glia and retinal vasculature in adult zebrafish**. A: Transverse view of peripheral retina in an adult *Tg(gfap:EGFP) *transgenic animal shows Müller cells (green) expanding through all retinal layers (blue: nuclear DAPI staining). B: Higher magnification shows Müller endfeet interposed with ganglion cell soma and contacting the retinal vessels (v). C: When the vascular layer is dissected from the inner interface of the retina (arrows) Müller endfeet remain attached to the vessels indicating a tight interaction. D: Blood vessel (green: Fli1-EGFP) overlying an adult retina seen from above with ganglion cell layer in the background (blue: DAPI nuclear staining). Müller cell endfeet (red: GFAP antibody) are observed on the entire surface of the inner retina, but especially concentrated along the retinal vessel, in direct contact with the vascular endothelium (yellow co-staining). *GCL: ganglion cell layer; v: vessel; IPL: inner plexiform layer; INL: inner nuclear layer; OPL: outer plexiform layer; ONL: outer nuclear layer*. Click here for file

Additional File 2 **Retinal vasculature in adult Plexin D1 mutants (*obd*) is characterised by a higher number of vessels and increased tortuosity**. A: Left and right retinas from an *obd *mutant showing 9–10 main vascular branches radiating from the optic disc. *Scale bar 1 mm*. B: Higher magnification of an adult *obd *retina exhibiting extraneous vascular branches and loops that are never observed in wild types. *Scale bar 200 μm*. C: Plexin D1 *obd *mutant retina exhibiting increased vessel tortuosity. *Scale bar 500 μm*. *OD: optic disc* Click here for file
