# Supplementary figures and images for: Genetic determinants of hyaloid and retinal vasculature in zebrafish
Source: BMC Dev Biol. 2007 Oct 15;7:114. doi: 10.1186/1471-213X-7-114 (PMC2169232; doi:10.1186/1471-213X-7-114)

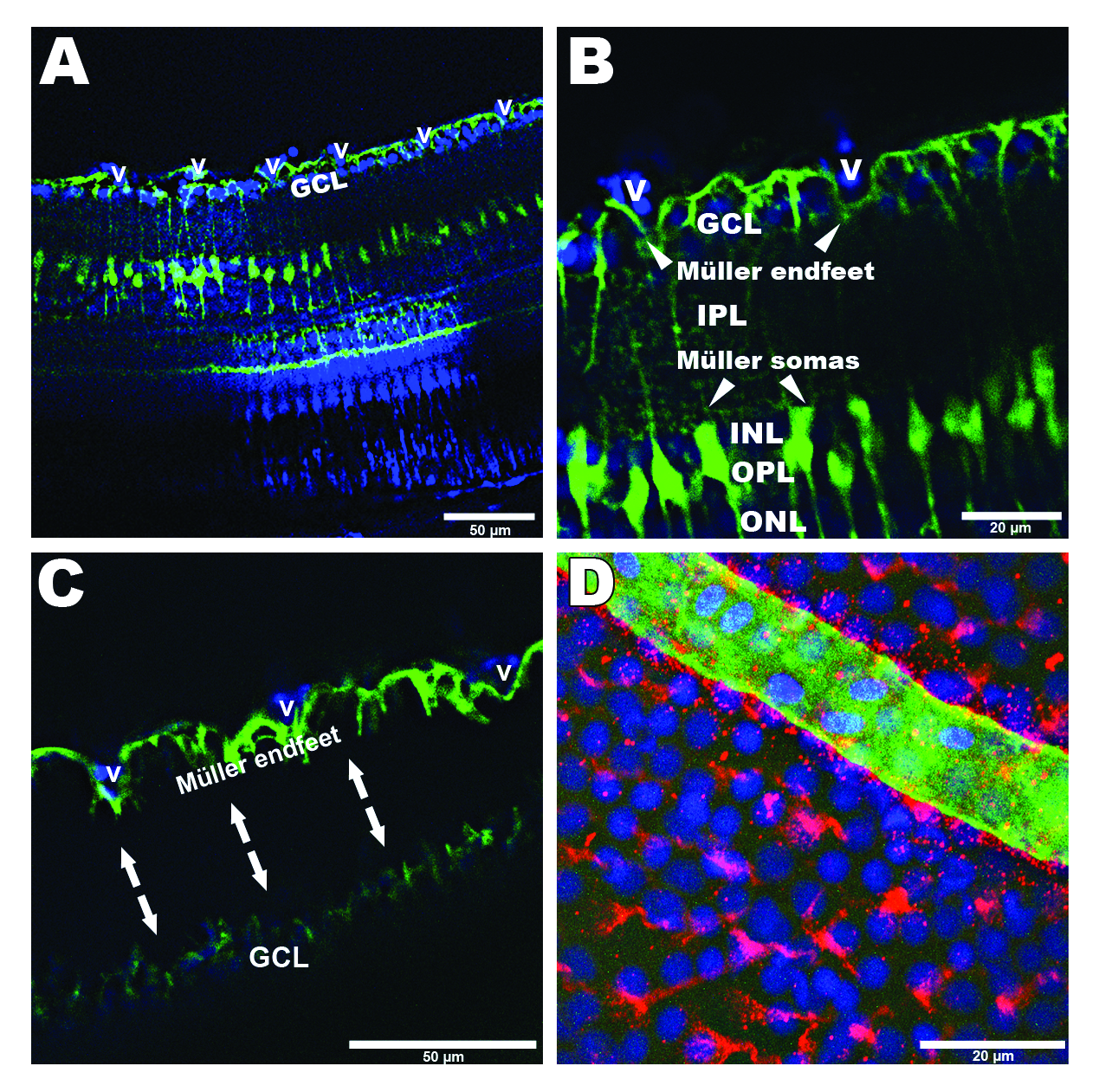

Supplement: Additional File 1 — Physical interaction of Müller glia and retinal vasculature in adult zebrafish. A: Transverse view of peripheral retina in an adult Tg(gfap:EGFP) transgenic animal shows Müller cells (green) expanding through all retinal layers (blue: nuclear DAPI staining). B: Higher magnification shows Müller endfeet interposed with ganglion cell soma and contacting the retinal vessels (v). C: When the vascular layer is dissected from the inner interface of the retina (arrows) Müller endfeet remain attached to the vessels indicating a tight interaction. D: Blood vessel (green: Fli1-EGFP) overlying an adult retina seen from above with ganglion cell layer in the background (blue: DAPI nuclear staining). Müller cell endfeet (red: GFAP antibody) are observed on the entire surface of the inner retina, but especially concentrated along the retinal vessel, in direct contact with the vascular endothelium (yellow co-staining). GCL: ganglion cell layer; v: vessel; IPL: inner plexiform layer; INL: inner nuclear layer; OPL: outer plexiform layer; ONL: outer nuclear layer. [file 1471-213X-7-114-S1.tiff]

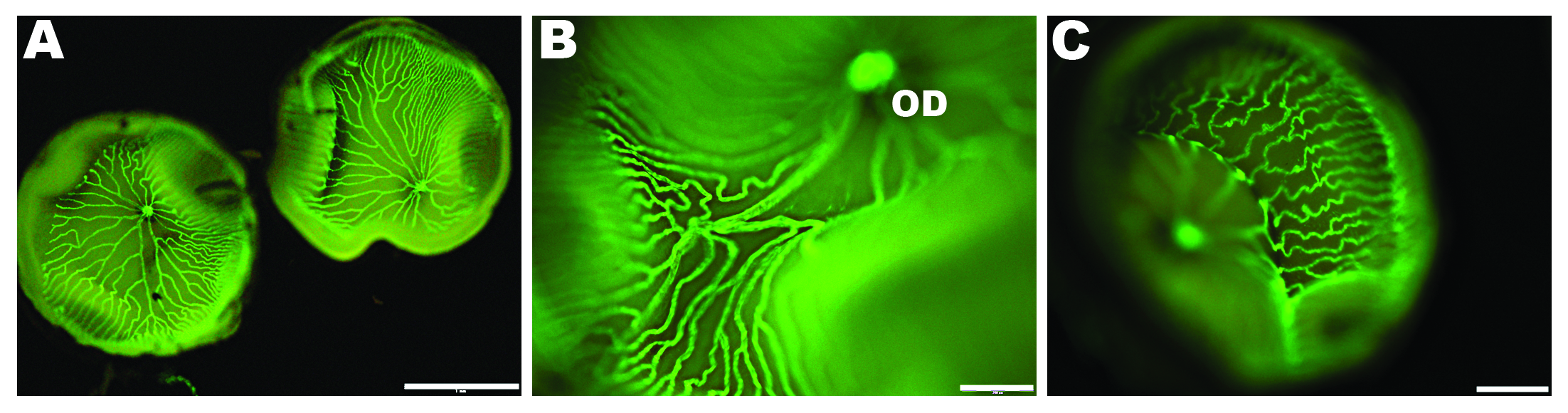

Supplement: Additional File 2 — Retinal vasculature in adult Plexin D1 mutants (obd) is characterised by a higher number of vessels and increased tortuosity. A: Left and right retinas from an obd mutant showing 9–10 main vascular branches radiating from the optic disc. Scale bar 1 mm. B: Higher magnification of an adult obd retina exhibiting extraneous vascular branches and loops that are never observed in wild types. Scale bar 200 μm. C: Plexin D1 obd mutant retina exhibiting increased vessel tortuosity. Scale bar 500 μm. OD: optic disc [file 1471-213X-7-114-S2.tiff]
